# Understanding How and by Whom COVID-19 Misinformation is Spread on Social Media: Coding and Network Analyses

**DOI:** 10.2196/37623

**Published:** 2022-06-20

**Authors:** Yuehua Zhao, Sicheng Zhu, Qiang Wan, Tianyi Li, Chun Zou, Hao Wang, Sanhong Deng

**Affiliations:** 1 School of Information Management Nanjing University Nanjing China; 2 Jiangsu Key Laboratory of Data Engineering and Knowledge Service Nanjing University Nanjing China

**Keywords:** health misinformation, COVID-19, social media, misinformation spread, infodemiology, global health crisis, misinformation, theoretical model, medical information, epidemic, pandemic

## Abstract

**Background:**

During global health crises such as the COVID-19 pandemic, rapid spread of misinformation on social media has occurred. The misinformation associated with COVID-19 has been analyzed, but little attention has been paid to developing a comprehensive analytical framework to study its spread on social media.

**Objective:**

We propose an elaboration likelihood model–based theoretical model to understand the persuasion process of COVID-19–related misinformation on social media.

**Methods:**

The proposed model incorporates the central route feature (content feature) and peripheral features (including creator authority, social proof, and emotion). The central-level COVID-19–related misinformation feature includes five topics: medical information, social issues and people’s livelihoods, government response, epidemic spread, and international issues. First, we created a data set of COVID-19 pandemic–related misinformation based on fact-checking sources and a data set of posts that contained this misinformation on real-world social media. Based on the collected posts, we analyzed the dissemination patterns.

**Results:**

Our data set included 11,450 misinformation posts, with medical misinformation as the largest category (n=5359, 46.80%). Moreover, the results suggest that both the least (4660/11,301, 41.24%) and most (2320/11,301, 20.53%) active users are prone to sharing misinformation. Further, posts related to international topics that have the greatest chance of producing a profound and lasting impact on social media exhibited the highest distribution depth (maximum depth=14) and width (maximum width=2355). Additionally, 97.00% (2364/2437) of the spread was characterized by radiation dissemination.

**Conclusions:**

Our proposed model and findings could help to combat the spread of misinformation by detecting suspicious users and identifying propagation characteristics.

## Introduction

### Background

As early as February 15, 2020, the General Director of the World Health Organization stated at the Munich Security Conference, “We are not only just fighting an epidemic; but also an infodemic” [1]. Owing to quarantine restrictions imposed during pandemics, information access is limited to the internet and social media, which facilitates misinformation spread. According to one survey, 87% of internet users were exposed to pandemic-related misinformation [2].

The spread of misinformation on social media can be amplified by information silos and echo chambers with personally tailored content. Kouzy et al [3] reported that 153 out of 673 tweets (24.8%) they examined contained COVID-19 pandemic misinformation, and 107 out of 673 tweets (17.4%) contained unverifiable information. Misinformation about the origin of the virus that originated from social media accounts has attracted more than 20 million hits [4].

### Theoretical Context

**Figure 1:**
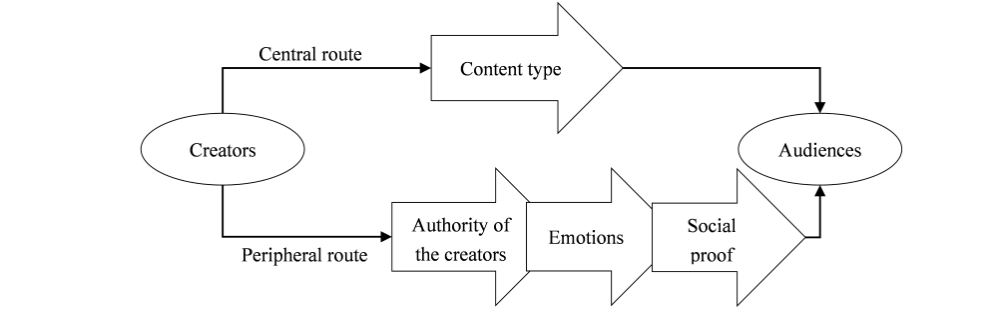
Theoretical model of the spread of COVID-19–related misinformation on social media.

On social media, misinformation can be defined as messages that aim to persuade other users. Persuasion theories state that the disseminator, message content, and recipient all have an impact on communication. Apart from studying the posts themselves, it is also necessary to examine the users who spread misinformation on social media. To uncover the characteristics of the spreaders of misinformation, we relied on persuasion theories that can help understand how misinformation is spread on social media. According to the elaboration likelihood model (ELM), a widely used persuasion model, users form their attitudes toward a message using either the central or peripheral path [5]. In the central path, users evaluate the quality and strength of the information, whereas in the peripheral route, they focus more on superficial factors such as the source’s reputation, visual appeal, and presentation [6]. Therefore, the characteristics of online health misinformation can be divided into two levels: central and peripheral [7]. The characteristics of the central-level features of online coronavirus misinformation have been documented in the context of various countries [8,9] with misinformation spreading rapidly worldwide. However, little is known about the peripheral characteristics of COVID-19–related misinformation. Therefore, the first research question of this study was as follows: What are the central- and peripheral-level features of disseminated misinformation related to COVID-19? To answer this question, we propose a theoretical model of the persuasion process of COVID-19–related misinformation on social media based on the ELM. As shown in Figure 1, the central route feature (content type) and peripheral features (including the creator’s authority, social proof, and emotion) are emphasized.

The dissemination of misleading information leads to increased public uncertainty, lack of belief in trustworthy sources, and, as a result, increased spread and ineffective containment of the virus [4]. To date, studies have largely focused on the extent of misinformation associated with coronavirus disease [3]. In contrast, a comprehensive understanding of the virality of COVID-19–related misinformation, particularly in the context of social media, is lacking. Accordingly, the second research question that guided this study was the following: How does COVID-19–related misinformation spread on social media? To answer this question, we measured the virality of COVID-19–related misinformation on social media from multiple perspectives, including the number of reposts, depth of reposts, width of reposts, and repost speed.

### Related Work

#### Persuasion Theory and Misinformation

Persuasion can be defined as “human communication that is designed to influence others by modifying their beliefs, values, or attitudes” [10]. The ELM views persuasion primarily as a cognitive event, meaning that the recipients of persuasive messages use mental processes of motivation and reasoning (or lack thereof) to accept or reject the messages [5].

In the peripheral route, messages rely on the emotional involvement of the recipient, and the recipient is persuaded by more superficial means. Cialdini [11,12] identified seven common cues that signal the use of a peripheral message: authority, commitment, contrast, liking, reciprocity, scarcity, and social proof. As a peripheral cue, authority can be used to convince the audience to accept the presented beliefs or behaviors. Previous work in psychology has shown that participants tend to believe information from people they consider credible [13]. From the social media perspective, the number of followers and followings (friends) of users may represent their social capital [14], which may indicate their authority. Posts by users with numerous followers (eg, opinion leaders) are perceived as trustworthy [15]. Moreover, Suh et al [16] found that the number of followers/followings of social media users positively influences the retweet probability of their posts.

Peripheral evidence of social proof is based on the age-old concept of peer pressure [11,12]. Empirical evidence suggests that engagement metrics provided by social media platforms, such as the number of likes and posts, also increase belief in social media content, especially in the case of misinformation [17]. Finally, rather than focusing on facts, the peripheral route depends on associations with positive attributes such as positive emotions [18].

#### Spread of Misinformation on Social Media

Bode and Vraga [19] defined misinformation as “the factually incorrect information that is not backed up with evidence.” Zhou et al [20] showed that misinformation with emotional and comparative terms is more likely to spread than correct information.

Moreover, researchers examined user-based characteristics to further understand the types of individuals who post or spread misinformation on social media [21]. Verification status, often assigned to official accounts and public figures to inform people that the account is authentic, is often used to measure the credibility of social media content [22]. Because crises are defined as emotional situations, the function of emotions in studies of misinformation connected to public health emergencies requires further investigation [23].

Existing research on the propagation characteristics of misinformation spread has focused on temporal factors [24,25] rather than propagation structure [26]. To capture the high-order propagation patterns of misinformation spread on social media, Ma et al [26] constructed a propagation network of misinformation on Twitter and identified that misinformation is typically first posted by a low-profile user, followed by some popular users who help spread it further, whereas genuine information is first posted by a prominent user and then directly shared by many general users.

#### Characteristics of COVID-19–Related Misinformation

Table 1 summarizes previous research on COVID-19–related misinformation on social media. Song et al [9] examined the types of misinformation disseminated during the COVID-19 pandemic in South Korea by analyzing fact-checking posts. Ceron et al [8] collected data from two Twitter accounts of Brazilian fact-checking projects and presented the refuted themes during the pandemic. Chen and Tang [27] reported that the spread of misinformation in public health emergencies had obvious characteristics of localization and high reproducibility.

In addition to content-based characteristics, the studies show that tweets from unverified accounts contain more misinformation compared to those from verified accounts (31% for unverified accounts, 12.6% for verified accounts; *P*<.001) [3]. Twitter accounts with more followers have fewer tweets with false information (20.1%, *P*<.001). Cinelli et al [28] found that while the number of posts from suspicious sources accounts for 70% of the volume of posts from trusted sources, the volume of responses to the former is three times higher than that to the latter.

In summary, there are two potential gaps in the existing literature that we address in this study. Previous studies have examined the characteristics of misinformation about the COVID-19 pandemic from several perspectives [3,8,9,27,29]. Psychological research has demonstrated the effect of news source credibility on persuasion [13], especially in the case of misinformation [30]. However, there is limited empirical research on the *source* of misinformation related to the COVID-19 pandemic (ie, the users who post the misinformation) and the misinformation dissemination patterns on social media platforms. In addition, based on the ELM, we proposed that the peripheral-level features of the spread of misinformation include the authority of the creators, emotion, and social proof, and then investigated the characteristics of the misinformation related to COVID-19 on social media. To the best of our knowledge, no study has considered all of the aforementioned features of COVID-19–related misinformation.

**Table 1:** Previous research on COVID-19–related misinformation on social media.

Study	Title	Method	Data	Source
Song et al [9]	The South Korean government’s response to combat COVID-19 misinformation: analysis of “Fact and Issue Check” on the Korea Centers for Disease Control and Prevention website	Content analysis	90 posts	Korea Centers for Disease Control and Prevention (KCDC) website
Kouzy et al [3]	Coronavirus goes viral: quantifying the COVID19 misinformation epidemic on Twitter	Statistical analysis	673 tweets	Twitter
Ceron et al [8]	Fake news agenda in the era of COVID-19: identifying trends through fact-checking content	Topic analysis	5115 tweets	Twitter
Qin [29]	Analysis of the characteristics of health rumors in public health emergencies: Taking the “Shuanghuanglian” incident during the COVID-19 as an example	Case analysis	134 headings	COVID-19–related rumor list announced by Dingxiangyuan.com
Chen and Tang [27]	Analysis of circulating characteristics of rumors on Weibo in public emergencies: a case study of COVID-19 epidemic	Coding and visual analysis	968 posts	Weibo Rumor Refuting

## Methods

### Process

**Figure 2:**
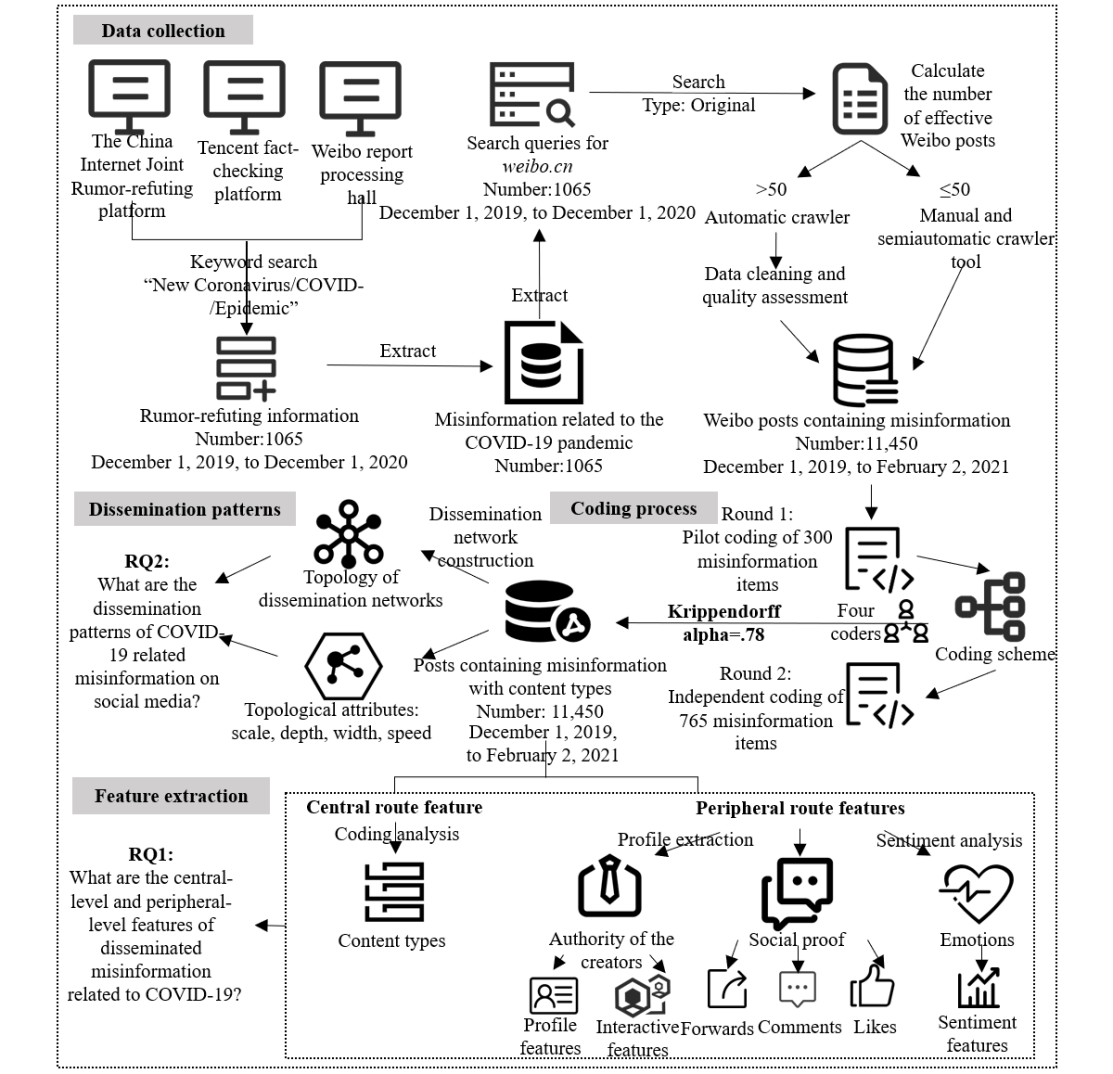
Data collection and data analysis process.

First, we created a data set of COVID-19 pandemic-related misinformation based on fact-checking sources and then created another data set of circulated posts that contained this misinformation from a real-world social media platform. Based on the collected posts, we further analyzed the dissemination patterns and proposed peripheral-level characteristics of the coronavirus misinformation circulated on social media. The detailed data collection and analysis procedures are described in Figure 2.

### Data Collection

#### Definition of Misinformation

Accurate identification of unknown misinformation is difficult for the general public because it requires multidisciplinary expertise. Reliable access to misinformation can be achieved by processing authoritative disconfirming information. For example, from the rebuttal information “Smoking can prevent coronavirus infection. This is false,” we can extract the misinformation “smoking can prevent coronavirus infection.”

#### Obtaining Misinformation Related to the COVID-19 Pandemic

As sources of authoritative misinformation, we selected three authoritative online platforms: China Internet Joint Rumor-Refuting Platform [31] (operated by the Office of the Central Cyberspace Affairs Commission), Tencent Fact Checking Platform [32] (operated by a large-scale internet integrated service provider), and Weibo Report Processing Hall [33] (operated by a large-scale public social media platform). We developed a web crawler to automatically collect the rumor-refuting information posted on each platform under the keywords “Novel Coronavirus (新冠病毒)/COVID/Epidemic (疫情)” from December 1, 2019, to December 1, 2020. After manual cleaning of irrelevant and repetitive information, we obtained 1065 COVID-19 pandemic–related misinformation posts.

#### Collecting Posts Containing COVID-19 Pandemic–Related Misinformation

To collect the circulated posts containing COVID-19 pandemic–related misinformation, we extracted keywords from all the collected misinformation, and then created corresponding queries for an advanced search on the Weibo.cn website. Considering the possibility of delayed and long-term dissemination of misinformation, the query search was limited to original posts between December 1, 2019, and February 2, 2021.

To ensure the accuracy of the collected posts, the first round of collection was performed by manual query using a semiautomated collection tool. If there were more than 50 valid retrieved posts, the second round of collection was performed using an automated web crawler followed by data cleaning. After two collection rounds, Weibo posts containing misinformation were matched with the corresponding misinformation and 11,450 posts were finally identified.

### Coding Process

A summary of previous research [34-36] on the classification of pandemic-related misinformation on social media can be found in Table S1 of Multimedia Appendix 1. Inspired by previous research, to characterize the content of COVID-19 misinformation, we manually coded the 1065 misinformation posts according to the six steps proposed by Richards and Hemphill [37]: (1) preliminary organization and planning, (2) open and axial coding, (3) development of a preliminary codebook, (4) pilot testing of the codebook, (5) final coding process, and (6) review of the codebook and finalization of the themes.

The coding scheme developed in this study is shown in Table 2. The COVID-19–related misinformation was classified into five content types: government response (Chinese-related), spread of epidemic (Chinese-related), medical information (Chinese-related), social issues and livelihood of people (Chinese-related), and international issues. Following Krippendorff’s [38] method, four coders were recruited to conduct two rounds of manual labeling. In the first round, a pilot labeling of 300 misinformation items was performed. After further discussion, the remaining 765 misinformation items were independently labeled by the four coders in the second round, with an α value of .78. This meant that the four coders achieved substantial agreement in the topic assignment [39]. In the coding process, for misinformation items involving two or more topics, all four coders discussed and classified the items into the most relevant categories. Subsequently, 11,450 posts were labeled according to the labeling of the corresponding misinformation.

**Table 2:** COVID-19 pandemic–related misinformation topics.

Topic	Illustration	Example
Government response (Chinese-related)	Information related to traffic control, resumption of work and school, suspension of work and school, epidemic prevention measures, and others	It is said that after the disinfectant powder is sprayed over Wuhan today, patients with fever will be transported to designated hospitals.
Spread of the epidemic (Chinese-related)	Information related to the spread of the pandemic	The son-in-law of the Guanghan family came back from Wuhan for a few days. The family concealed their working address and went to play cards every day. He became ill today. The neighbors were very angry and went to smash his house.
Medical information (Chinese-related)	Information related to the virus itself, infection, prevention, treatment, disinfection, and other medical information	A doctor friend sent it. In response to this new type of coronavirus, the content of vitamin C (to fight the virus) and echinacea (to enhance immunity) can be used to prevent it.
Social issues and livelihood of people (Chinese-related)	Information related to celebrities, donation assistance, social aspects, and people’s livelihood	National level response! All rented houses, apartments, shops and factories will be rent-free for one month in February, and rent-free for half a month in March and April! I hope that all “landlords” will respond positively! Overcome the difficulties together
International issues	Information related to other countries’ response, online political rumors	Japan sent a 1,000-member medical team to Wuhan without masks and slogans.

### Extraction of Post Features

Through the weibo.cn/repost/ website using a Weibo ID, we obtained the specific forwarding, liking, and commenting information of each post. Based on the forwarding relationships, we then created the forwarding network of the collected posts. Following Avram et al [17], we used engagement metrics, including the numbers of likes, comments, and forwards of each post, to represent the social proof features of posts containing misinformation (as summarized in Table 3).

**Table 3:** Features of posts containing COVID-19–related misinformation and users who have posted them.

Category		Description	Data type
**Social proof features**			
	Forwards	Frequency of forwarding	Integer
	Comments	Frequency of commenting	Integer
	Likes	Frequency of liking	Integer
**Profile features**			
	Verification status	Verified or not	Verified/Not verified
	Verification type	Verification type	Category
	Mrank	Weibo membership level	Integer (0-7)
	Urank	User level	Integer (0-48)
**Interactive features**			
	Posts_count	Number of posts	Integer
	Followers_count	Number of followers	Integer
	Following_count	Number of followings	Integer

### Extraction of User Features

Apart from users who could not be captured because they were, for example, blocked, a total of 11,301 users who had published posts containing misinformation about COVID-19 were collected on Weibo.

Weibo’s user authentication mechanism provides a channel for different types of users to prove their identity. The type of verification includes verified personal users, government users, media users, and businesses. The user level, as the basic characteristic of Weibo users, can largely represent the activity level of accounts. The higher the user level, the more active the user is. Membership level reflects users’ habits in using Weibo. Users with a high membership level can be considered loyal users.

In addition to profile features, the interactive characteristics (ie, numbers of followers, followings, and posts) can also characterize users’ authority on social media. The number of posts reflects the user’s engagement on the social media platform. Users with a considerable number of followers can share their opinions with a large group of people [16], whereas users with many followings have a broad range of information sources [14]. Therefore, both profile features and interactive features characterize the authority of users who have published posts containing misinformation. Detailed descriptions of these features can be found in Table 3.

### Sentiment Analysis

Sentiment characteristics have been recognized as effective features for distinguishing online rumors and fake reviews [40]. In this study, we performed sentiment analysis by applying a pretrained convolutional neural network model to the collected data set on the Baidu Senta system [41]. Senta incorporates sentiment knowledge into pretrained models and produces new state-of-the-art results on most of the test data sets [42]. Senta is exposed to a vast corpus from multiple settings as an open sentiment analysis platform, which considerably increases the validity of its analyses [42,43]. Senta uses an unsupervised method to automatically mine emotional knowledge, and comprehensively surpassed other approaches in 14 typical tasks of Chinese emotional analysis [41]. Each post that is input into Senta returns an outcome of positive sentiment likelihood ranging from 0 to 1, which can be utilized as the sentiment feature of the post.

### Construction of the Dissemination Network

To describe the prevalence of coronavirus-related misinformation on social media, in addition to the number of forwards of each post, we crawled the detailed forwarding information for each post in the data set created in the previous step of the research and collected a list of forwards of the original misinformation posts. The forwarding information for each post included the users who forwarded the original post, the content of the reposts, and the forwards and likes that the reposts received. The Weibo platform uses the “//” symbol to divide the forwarded content into different forwarding levels. Therefore, the forwarding level of each post can be extracted based on the forwarded content. In addition, the dissemination network of each post can be constructed using a series of reposts based on the corresponding forwarding relationships. Thus, apart from posts that could not be captured because they were, for example, blocked or deleted, we constructed a dissemination network for a total of 2437 posts that contained information about COVID-19. In these networks, each node represents an individual post, whereas a directed link represents a forwarding relationship from the source node to the repost node. For example, if post A forwards the original post B, then an edge is drawn from nodes B to A.

### Extraction of Dissemination Scale, Depth, Width, and Speed

In the constructed dissemination networks, each node represents a single post that was involved in the spread of misinformation related to COVID-19. Based on the network for each original post, the dissemination scale refers to the number of nodes in the network, corresponding to the number of forwards for the original post. The dissemination depth indicates the highest level of repost in the network of the original post, whereas the dissemination width is equal to the number of nodes at the level with the largest number of nodes in the network.

**Figure 3:**
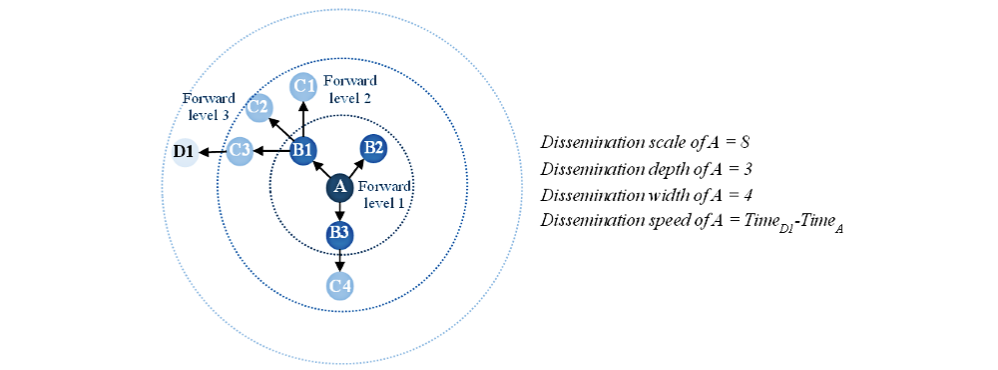
Illustration of the dissemination scale, depth, width, and speed of a sample post. Each node represents a single post that was involved in the spread of misinformation related to COVID-19.

Figure 3 shows an example of a dissemination network for a post. Node A denotes an original post containing COVID-19–related misinformation. Posts B1, B2, and B3 forward the original post. Subsequently, post B1 is forwarded by posts C1, C2, and C3, while post B3 is forwarded by post C4; post C3 is also forwarded by D1. In this case, the original post A has been forwarded eight times and thus has a dissemination scale of 8. The second level of forwarding involves most posts, which means that the propagation width of the original post A is equal to 4. The highest level of forwarding signifies that the dissemination depth is 3.

### Ethical Considerations

No ethics approval was required as this study was based on publicly available data and involved no personally identifiable data.

## Results

### Central Route Feature of Posts

To answer the first research question, coding analysis was performed to identify the content types/topics of posts containing COVID-19–related misinformation. A total of 11,450 such posts were categorized under five topics: government response (n=1021), spread of the epidemic (n=639), medical information (n=5359), social issues and livelihood of people (n=4132), and international issues (n=299). The most common theme was medical misinformation (5359/11,450, 46.80%), including misinformation about the virus, infection, prevention, treatment, and disinfection. The second most popular topic was social issues and livelihood of people (4132/11,450, 36.09%), especially related to fake statements about celebrities. This category also included posts referring to donations that were refuted.

**Figure 4:**
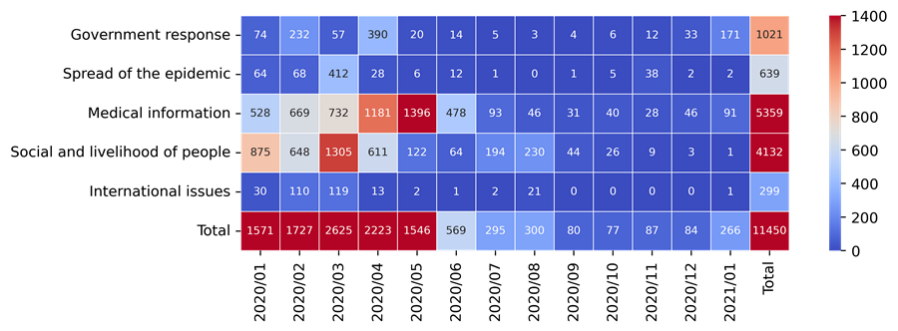
Changes in the number of posts containing misinformation over time.

To distinguish different topics of posts, the number of posts and corresponding dates are plotted in Figure 4, where the warmer color represents a more popular misinformation topic in a given period. Social and livelihood misinformation emerged most prominently in mid-March when several widely circulated misinformation topics appeared, including statements about a Malaysian shaman who can cast a spell to cure the coronavirus. Posts with misinformation about medical information appeared most frequently from February to May, which coincided with the most severe period of the epidemic in China.

### Peripheral Route Features of Posts

#### Overview

To answer the first research question, we also examined the social proof features of the collected posts, the sentiment features of the posts, and the authority features of the users who posted them.

#### Social Proof Features of the Posts

**Figure 5:**
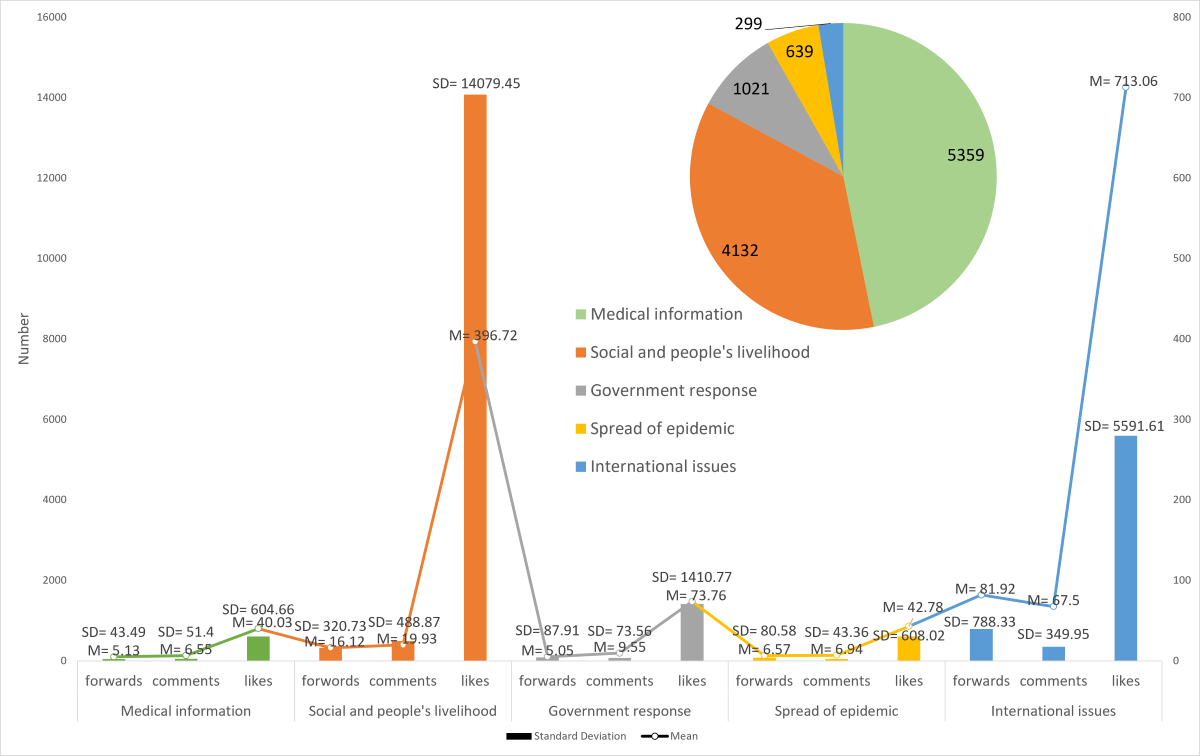
Social proof features of posts related to various misinformation topics.

The collected posts with COVID-19–related misinformation received 11 forwards, 13 comments, and 189 likes, on average. The pie chart in Figure 5 shows the number of posts containing misinformation on different topics, and the bars and the line show the standard deviations (on the left Y-axis) and average numbers (on the right Y-axis) of likes, comments, and forwards that the posts on each topic received. Considering the topics of misinformation, posts with the most likes (mean 713), comments (mean 67), and forwards (mean 82) contained misinformation on international issues, whereas posts with medical information received the least attention, with an average of 40 likes, 7 comments, and 5 forwards (see Figure 5). Among all five topics, posts containing misinformation on international issues accounted for only 2.61% (299/11,450) of all posts, but achieved much higher attention. This may suggest that misinformation involving international issues, while less frequent, is much more viral across all three types of social proof features, indicating the potentially serious consequences of such misinformation.

#### Sentiment Features of the Posts

**Figure 6:**
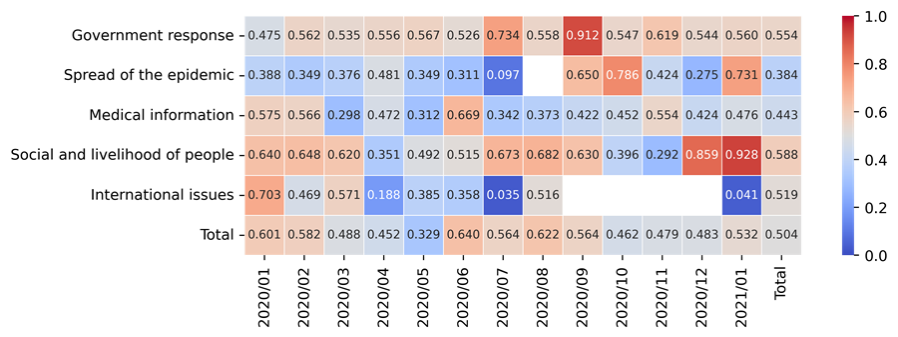
Sentiment features of posts related to various misinformation topics.

Considering the misinformation topics, Figure 6 describes the variance of the mean value of the generated sentiment intensity value of the COVID-19-related misinformation posts. The warmer the color, the more positive the emotion, and conversely, the colder the color, the more negative the emotion. The average sentiment value of the posts remained neutral to positive over time. Some of the posts that contained misinformation about unproven preventive measures and treatments appeared to be relatively positive. For example, “A hot bath in the home is the easiest, most effective, and least costly way to protect susceptible people!” conveyed extremely optimistic emotions. In contrast to the general negative sentiment that prevailed among the public during the pandemic [44], posts containing misinformation tended to express positive emotions to attract public attention.

In contrast to other topics, posts spreading misinformation related to spread of the epidemic tended to be consistently negative. In particular, misinformation related to the lifting of the lockdown and traffic restrictions expressed very negative emotions, such as “Harbin is closed! Urgent city closure. No chance for any travel.”

#### Authority Features of Users

Among the users who posted messages containing misinformation related to COVID-19, certified users accounted for 46.60% (5266/11,301). Among them, verified personal users were the most prominent sources (2475/5266, 47.00%), followed by media users (1159/5266, 22.01%) and government accounts (1013/5266, 19.24%). The number of nonverified users was only 6.8% more than that of verified users, representing 53.40% (6035/11,301) of the messages. This suggests that when detecting misinformation, whether it was published by a verified account cannot be a criterion for determining the authority of the information.

We found obvious differences between the authority characteristics of users who posted messages containing misinformation on different topics. For international issues, certified users (160/292, 54.8%) outnumbered uncertified users (132/292, 45.2%), and certified users (3160/5310, 59.51%) also outnumbered uncertified users for medical information. By contrast, misinformation about social issues and people’s livelihoods was more likely to be posted by uncertified users (2586/4093, 63.18%) than by certified users (1507/4093, 36.82%), and misinformation about the spread of the epidemic was also more likely to be posted by uncertified users (409/630, 64.9%).

**Figure 7:**
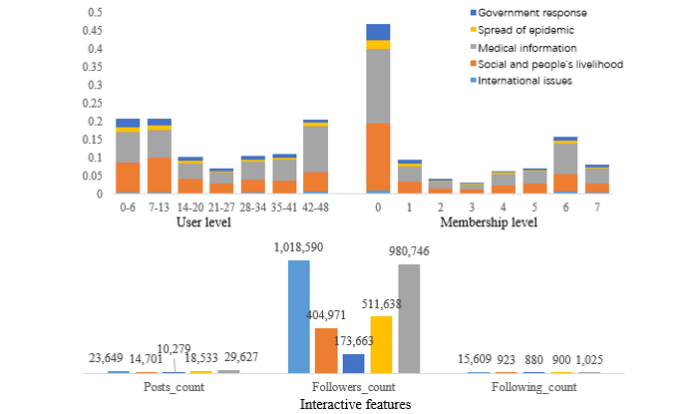
User-level and membership-level distributions and average interactive features of users posting misinformation about various topics.

The user-level and membership-level distributions of users posting misinformation on various topics are shown in the upper part of Figure 7. Considering the user and membership levels, the users with the lowest levels (in the range of 0-13 for user level and 0 for membership level) were the most responsible for publishing misinformation, accounting for 41.24% (4660/11,301) and 46.77% (5285/11,301), respectively. Surprisingly, the second most responsible user groups were those with the highest user (in the range of 42-48; 2320/11,301, 20.53%) and membership (in the range of 6-7; 2680/11,301, 23.71%) levels. This may indicate that misinformation is posted by both the least and most active users. For international misinformation and medical misinformation, users with the highest user levels (in the range of 42-48) accounted for the most (1516/5602, 27.06%), while misinformation on the other three topics tended to be posted more by users with lower user levels (in the range of 0-13; 2758/5699, 48.39%).

The lower part of Figure 7 shows the average interactive features of users who posted messages containing misinformation on different topics. The number of followers of misinformation publishers averaged over 100,000, demonstrating the great social capital they have on social media.

In comparison, users who posted misinformation related to international issues and medical information tended to have higher authority than those who posted about the government response, social issues and livelihood of people, and spread of the epidemic. The numbers of posts, followers, and followings of users who posted misinformation related to the government response were the lowest among the five topics, representing users who had less authority.

### Dissemination Patterns of the Posts

Based on the constructed dissemination network for each of the 2437 posts containing COVID-19–related misinformation, we extracted the dissemination scale, depth, maximum width, average width, and speed for each post. In this study, maximum width measured the number of nodes involved in the widest level and average width measured the average number at all levels. Table 4 summarizes the descriptive statistics for the dissemination patterns of all posts. In our data set, the most widely disseminated post comprised 7604 users in the dissemination network. The posts that spread the deepest were forwarded by 14 levels of users, whereas the posts that spread the most widely were forwarded by 2355 users in a given dissemination level. On average, the dissemination scale of all posts was 19.7, with an average depth of 1.5 and an average maximum width of 20.5.

**Table 4:** Descriptive statistics of the dissemination patterns.

Dissemination measures	Mean (SD)	Maximum
Scale	19.7 (236.03)	7604
Depth	1.5 (0.99)	14
Maximum width	20.5 (87.82)	2355
Average width	15.9 (23.74)	688
Speed	2.4 (8.20)	96.9

**Figure 8:**
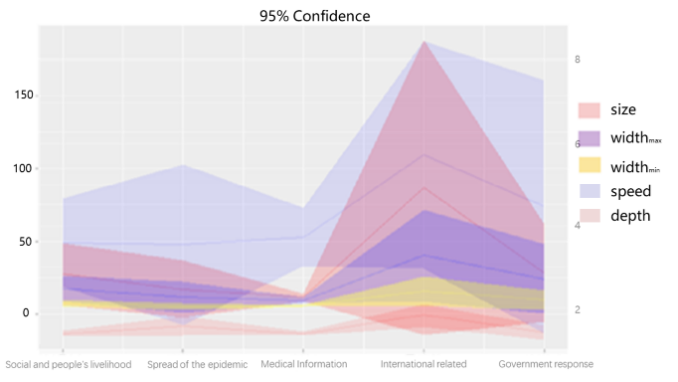
Confidence interval plots for the dissemination patterns.

Figure 8 shows the 95% CI plots for the means of the dissemination scale, depth, maximum width, average width, and speed for the posts related to each topic. Posts containing misinformation related to social issues and livelihood of people clearly engaged many more users in the dissemination network than posts on other topics. Compared to the other topics, misinformation related to the government response (average dissemination scale 28.2) and social issues and livelihood of people (average dissemination scale 27.4) engaged more users in the discussions and propagation. In terms of dissemination depth, maximum width, and average width, posts on international issues reached a larger audience at each dissemination level and resulted in more in-depth propagation. Unlike other topics, most international misinformation posts attracted some level of public attention (with a forwarding rate of 168/299, 56.2%), suggesting that this type of misinformation is more likely to be subject to widespread, large-scale, and heated mainstream discussions.

**Figure 9:**
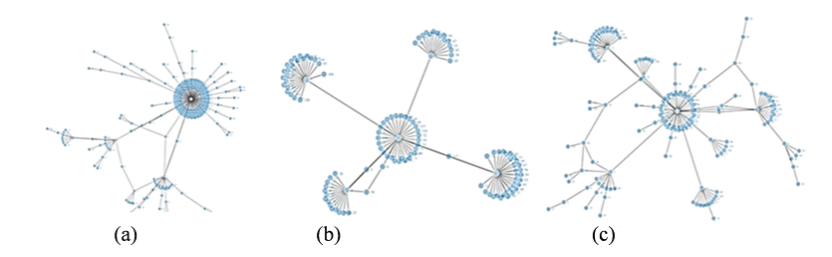
Examples of each dissemination network type. (a) Radiation dissemination network. (b) Sector dissemination network. (c) Viral dissemination network.

Based on the structure, we divided the dissemination network of the misinformation posts into three main types: (1) radiation dissemination network, where the first-level dissemination is wider than all other levels; (2) sector dissemination network, where the width of the other levels in the dissemination network is wider than that of the first level, and the node with the highest forwarding volume reaps more forwards than likes; and (3) viral dissemination network, where the width of other levels in the dissemination network is larger than that of the first level, and the node with the highest like volume reaps more likes than forwards. Figure 9 shows representative examples of all three networks.

Examination of the posts revealed that 97.00% (2364/2437) were disseminated through the radiation dissemination network, only 0.98% (24/2437) belonged to the sector dissemination network, and 2.01% (49/2437) belonged to the viral dissemination network. As shown in Figure 9a, the width of the first-level forwarding radiated from the original node is much larger than that of the subsequent layers, which is reflected in the much higher density of nodes around the root node than that of nodes on the other levels. In this type of dissemination network, the user who created the original post usually has higher authority (eg, a large number of followers); such users include public organizations and news media. In addition, the content of the posts is likely political. The node density tends to decrease as the level increases, indicating that the potential impact of these posts declined after the first-level forwarding. For the dissemination networks shown in Figure 9b and c, although the width of the first level is relatively large, the nodes at the other levels are fan-shaped, with the nodes radiating from multiple sublevel nodes. In the spread of posts pertaining to the sector and viral dissemination networks, there are multiple nodes with a high degree of propagation ability. In contrast to the posts present in the radiation dissemination network, some opinion leaders, who possessed a large number of followers and were not verified as authoritative institutions or public organizations, also played a role in the spreading of the posts in the sector and viral dissemination networks.

### Clustering Analysis of Misinformation Disseminators

**Figure 10:**
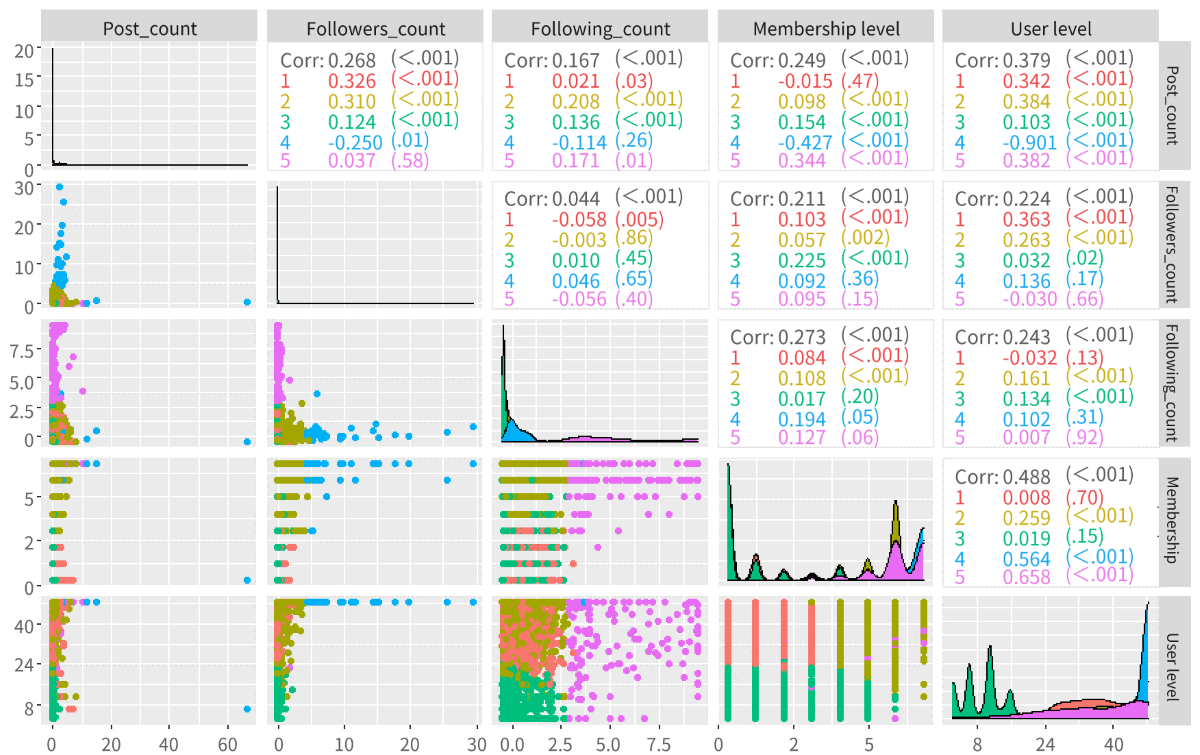
Scatterplot and correlation matrix of user authority features. a: The correlation is significant at a significance level of .001 (two-sided); b: The correlation is significant at a significance level of .01 (two-sided); c: The correlation is significant at a significance level of .05 (two-sided).

To further characterize the users who posted misinformation about coronavirus on social media, we leveraged the k-means clustering algorithm to classify users based on user authority features (including user level, membership level, posts count, follower count, and following count). To ensure the quality of the clustering, the Nbclust function in R was used to test different values of k. Based on the elbow method, 5 was selected as the optimal number of clusters. Figure 10 shows the users in the form of a scatterplot matrix created using the ggpairs function from the GGally R package [45]. Each point is colored by a cluster identified using the k-means clustering algorithm.

As determined by the k-means clustering algorithm, users who posted misinformation were classified into five groups: general users, platform users, inactive users, influential users, and minglers. A total of 2342 users were classified as general users, who participate in social media but are less willing to pay for membership. They tended to have a low membership level but a high user level, and their performance in terms of the number of posts and followers was relatively normal. The behavioral patterns of platform users (comprising 2980 users) were similar to those of general users, except for their membership level. They tended to have significantly higher membership levels, indicating that they were both actively participating in social interactions and purchasing memberships to enjoy the privileges. The largest group, inactive users, comprised 5652 users who appeared at lower frequencies for all five features. In contrast, influential users, the smallest group of users (comprising 101 users), posted more frequently than others and also reaped a large number of followers. Users in this group tended to remain in the highest position with respect to both user and membership levels. Finally, users in the mingler group had a higher number of followers than the other user groups, but they made fewer posts. The characteristics of the 226 users in the mingler group were consistent with those identified by Kozinets [46] as users who maintain strong social ties on social media while being marginally interested in activities.

**Figure 11:**
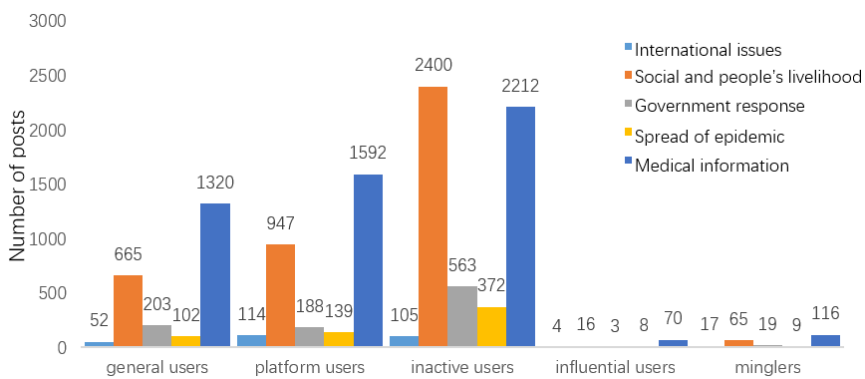
Distribution of various types of users posting misinformation related to various topics.

The distribution of different types of users posting misinformation on different topics is shown in Figure 11. Inactive users were more likely to post misinformation about social issues and livelihood of people, which significantly differed from the other four user types. Influential users contributed the most to medical misinformation dissemination, but were less likely to post misinformation related to social issues and people’s livelihoods.

### Correlations Between Topological Attributes and User Authority Features

We also performed a correlation analysis to test whether the dissemination network features were significantly related to the authority features of the users who created the posts. The Spearman rank correlation coefficient was used to measure the correlations between the authority features of the creators and the features of the resulting dissemination network. Table 5 shows that the number of followers and membership level were significantly correlated with all five characteristics of the dissemination network (ie, dissemination scale, depth, average width, maximum width, and speed) under two-sided test conditions (*P*<.01).

From a network perspective, messages posted by users with numerous followers tend to receive more attention on social media. Users with high membership levels are more likely to engage in social media interactions. Similar to the context of disaster-related information [14], the number of followers was found to have a significant positive effect on the dissemination scale, depth, and speed of the post. This suggests that misinformation from users with high authority levels have a high intensity in wide broadcasts, and thus can have serious consequences.

**Table 5:** Spearman correlation (*ρ*) analysis between topological attributes and user authority features.

Dissemination variables			Posts count		Followers count		Following count		Membership level		User level
**Scale**											
	*ρ*	0.114		0.344		0.009		0.171		0.107	
	*P* value	<.001		<.001		.77		<.001		.001	
**Maximum width**											
	*ρ*	0.103		0.349		0.008		0.17		0.1	
	*P* value	.002		<.001		.80		<.001		.002	
**Average width**											
	*ρ*	0.081		0.345		–0.007		.171		0.096	
	*P* value	.01		<.001		.83		<.001		.003	
**Depth**											
	*ρ*	0.174		0.197		0.106		0.08		0.118	
	*P* value	<.001		<.001		.001		.02		<.001	
**Speed**											
	*ρ*	0.023		0.174		–0.003		0.105		0.047	
	*P* value	.48		<.001		.93		.001		.16	

## Discussion

### Principal Findings

Understanding the underlying psychology of why people fall for misinformation is key to developing effective interventions against it [30]. This study of posts containing COVID-19–related misinformation reveals important insights into its dissemination on social media. To analyze the central-level feature, the COVID-19–related misinformation content was classified into five types: medical information (5359/11,450, 46.80%), social and livelihood of people (4132/11,450, 36.09%), government response (1021/11,450, 8.92%), spread of the epidemic (639/11,450, 5.58%), and international issues (299/11,450, 2.61%). The classification of the content is consistent with previous research on recent epidemics [34]. Consistent with previous studies on COVID-19 in which most of the misinformation was medical-related [1,3], we found that the predominant themes of misinformation were related to medical information, as well as to social and livelihood issues. In particular, posts with medical misinformation appeared most frequently during the most severe phase of the pandemic.

Misinformation that attracts attention can trigger intense discussions, thus promoting the spread of information. In addition to the central-level feature, social proofs of posts with COVID-19–related misinformation showed that such misinformation was actively responded to (with an average of 11 forwards, 13 comments, and 189 likes). Interestingly, misinformation related to international issues accounted for 2.61% (299/11,450) of all posts but achieved alarmingly higher attention (with an average of 82 forwards, 67 comments, and 713 likes), suggesting that misinformation involving international issues tends to go viral on social media and thus can have serious consequences. This is consistent with empirical findings on Twitter, where COVID-19–related conspiracy misinformation is most likely to spread [47].

In contrast to the negative sentiment that emerged among the public during the pandemic [48], positive to neutral sentiment in the misinformation posts appeared during the pandemic. In particular, some misinformation about the untested prevention measures and treatment seemed extremely positive and captured the public’s attention. Our results support the idea that misinformation topics should be considered when designing misinformation interventions during the pandemic [47].

Analysis of user profile characteristics revealed that users with the lowest and highest levels of user and membership levels were the most responsible for publishing misinformation. Our results suggest that both the least and most active users are prone to sharing misinformation. In contrast to the empirical results of Kouzy et al [3], where unverified Twitter accounts published significantly more misinformation than verified accounts, verified Weibo users accounted for nearly half (5266/11,301, 46.60%) of all messages containing COVID-19–related misinformation. This suggests that user verification status is not applicable to coronavirus-related misinformation detection on Weibo.

The average number of followers of the misinformation publishers was extremely high (>100,000), indicating the credibility and social influence they possess on social media. Some marketing-oriented accounts changed the main part of genuine news to attract users. As for the medical misinformation, some corporate accounts fabricated misinformation (eg, “Natto can inactivate the virus”) to promote their product.

The average dissemination scale of misinformation posts was 19.7, with an average depth of 1.5 and an average maximum width of 20.5. Li et al [14] reported average dissemination scale and depth for disaster-related posts of 68.64 and 1.113, respectively. This suggests that COVID-19–related misinformation posts spread deeper than disaster-related posts, while the dissemination scale was lower than that of disaster-related posts. Moreover, posts about international issues were the most likely to have profound and lasting effects on social media, with the highest numbers in terms of dissemination depth, maximum width, and average width. This highlights the importance of dealing with COVID-19–related misinformation, especially that related to international issues.

In capturing the topological attributes of the dissemination network, three main types of networks can be distinguished in the spread of misinformation posts: radiation, sector, and viral. Unlike rumor-spreading on Twitter, in which the news is usually first posted by a low-impact user and then shared by some popular users [26], the majority of COVID-19 misinformation on Weibo was represented by the radiation dissemination network, in which the messages were first posted by a prominent user and then directly shared by many general users. In addition, the original user tended to have higher authority (public organizations and news media), suggesting the crucial role of influential users in the spread of COVID-19 misinformation; this is consistent with the results of Wang et al [47], who found that Donald Trump’s tweets potentially influenced people’s information-sharing behavior.

### Limitations

This study has several limitations. First, we only examined misinformation about COVID-19 circulating on Weibo. In addition, we selected “Novel Coronavirus (新冠病毒)/COVID/Epidemic (疫情)” as COVID-19–related keywords. However, due to the potential early inconsistency in disease terminology, users may have used other keywords that were not collected by this study, such as 武汉肺炎 (Wuhan pneumonia) and 不明原因肺炎 (unknown-cause pneumonia), to describe COVID-19–related conversations or topics. Therefore, the characteristics identified in our study may not represent all COVID-19–related misinformation. Future studies should consider misinformation on other social media platforms to ascertain the stability of these findings. Second, we focused on Chinese-language misinformation. Misinformation in other languages about the pandemic could lead to different results, which should also be explored in future work.

### Conclusions

In the COVID-19 pandemic, we witnessed a massive infodemic in which fake news and conspiracy theories were spread, especially on social media. This study provides a comprehensive examination of the COVID-19 misinformation spread on a social media platform.

The theoretical contributions of this study lie in the following two aspects. Although efforts have been made to analyze the COVID-19–related misinformation on social media platforms, no comprehensive analytical framework guided by psychological theory exists to study such misinformation, particularly related to COVID-19. Based on the ELM, this work provides a first step toward understanding the underlying persuasion process of COVID-19–related misinformation. By developing a theoretical model of the persuasion process, this study includes a comprehensive set of features to understand the spread of COVID-19–related misinformation on social media. Moreover, whereas previous studies have generally considered the detection of pandemic misinformation as a binary classification problem, our results show that misinformation on different topics appears to have different characteristics in terms of emotion, social engagement metrics, and publisher authority characteristics. Therefore, this study suggests that the development of misinformation detection algorithms and prevention mechanisms should consider the specific topics of misinformation. It is necessary to develop targeted strategies based on the characteristics of misinformation on different topics.

The practical contributions of this study are two-fold. First, although COVID-19–related misinformation has been widely studied, to our knowledge, no research has attempted to uncover the comprehensive characteristics of users who post misinformation about the novel coronavirus on social media. Therefore, this study examined both the profile features and the interactive characteristics of misinformation authors. By revealing the characteristics of misinformation publishers, our results not only extend the research on analyzing COVID-19–related misinformation but also provide a possible solution to the issue of detecting suspicious users who may be prone to posting misinformation. Moreover, the significant positive correlations among the authority features of the users and the topological attributes of the dissemination network indicate the possible influence of authority features on the spread of misinformation. To combat misinformation, our results suggest that it is important for influential users, public organizations, and news media to be aware of their responsibility to provide verified information, especially during a public health crisis.

## Appendix

Multimedia Appendix 1 Table S1. Summary of previous research on the classification of coronavirus-related misinformation on social media.

## Abbreviations

ELM: elaboration likelihood model
