# Supplementary material for: Understanding How and by Whom COVID-19 Misinformation is Spread on Social Media: Coding and Network Analyses
Source: J Med Internet Res. 2022 Jun 20;24(6):e37623. doi: 10.2196/37623 (PMC9217148; doi:10.2196/37623)
Supplement: Multimedia Appendix 1 [file jmir_v24i6e37623_app1.docx]

Multimedia Appendix 1:

Table S1. A summary of previous research on the classification of coronavirus-related misinformation on social media.

| Study | Classification |
| --- | --- |
| Hu, Chen [1] | Prevention and treatment |
|  | Social and people’s livelihood |
|  | Medical information |
|  | Traffic control |
|  | Epidemic spread |
|  | Celebrity related categories |
|  | Conspiracy theory |
|  | Rescue |
| Wang [2] | Misunderstanding of virus |
|  | False epidemic information |
|  | Fabricated impact of the epidemic |
|  | Fabricate prevention and control measures |
|  | Rumors of special characters |
|  | Other |
| Yao, Ma [3] | Medical information |
|  | Lifestyle |
|  | Education |
|  | Location |
|  | Social information |

Reference:

1. Hu W, Chen H, Wang Q. Psychological motivations and countermeasures of rumors in COVID-19 epidemic. Journal of Dali University. 2021;6(1):111-6. doi: 10.3969/j.issn.2096-2266.2021.01.018.
2. Wang K. Content analysis of rumors related to the Novel Coronavirus epidemic and reflections on governance--Analysis of 368 samples based on Nvivo 11. Journal of Northeast Agricultural University (Social Science Edition). 2020;18(05):27-35.
3. Yao A, Ma J, Lin Y, Liu Q, Zhang Z. The rumor evolution and governance strategy of major public health emergencies. Information Science. 2020;38(07):22-9. doi: 10.13833/j.issn.1007-7634.2020.07.004.
